# Physical Activity Frequency and Depression in the Spanish Population

**DOI:** 10.3390/ijerph192214704

**Published:** 2022-11-09

**Authors:** Ángel Denche-Zamorano, David Ajenjo-Gomez, Damián Pereira-Payo, Carmen Galán-Arroyo, Alejandro Vega-Muñoz, Nicolás Contreras-Barraza, Miseldra Gil-Marín, Jorge Perez-Gomez

**Affiliations:** 1 Promoting a Healthy Society Research Group (PHeSO), Faculty of Sport Sciences, University of Extremadura, 10003 Caceres, Spain; 2 Instituto de Investigación y Postgrado, Facultad de Ciencias de la Salud, Universidad Central de Chile, Santiago 8330507, Chile; 3 Facultad de Economía y Negocios, Universidad Andrés Bello, Viña del Mar 2531015, Chile; 4 Public Policy Observatory, Universidad Autónoma de Chile, Santiago 7500912, Chile; 5 Health Economy Motricity and Education (HEME) Research Group, Faculty of Sport Science, University of Extremadura, 10003 Caceres, Spain

**Keywords:** depression, exercise, strength, phq-8, mental health

## Abstract

Introduction: Depression is a concerning mental health disorder. It is the first cause of inability worldwide, which entails high economic costs for the public system. Current evidence suggests that physical activity is an effective tool for the prevention and treatment of depressive symptoms. Objective: To examine the relationship between the cases of depressive symptoms and depression presented by the adult Spanish population and the days per week of physical activity and strength training that they perform. Design: It is a cross-sectional study with data from the European Health Survey of Spain 2020 (EESE 2020), consisting of 10,024 males and 11,126 females, between 18 and 84 years, which conformed the sample of 21,150 participants. Results: Depressive symptoms and depression were related to days of PA per week, PA frequency per week and strength training days per week (*p* < 0.001), depressive symptoms and depression were found to have higher prevalence in the inactive groups than in the active groups (*p* < 0.05); this phenomenon was found in both sexes and age groups. Conclusions: The prevalence of depressive symptoms and depression were associated with physical activity in the general population, by sex and by age group too. The prevalence of both were higher in the inactive population than in the active population of all ages and sexes. Future studies are required to confirm the relationship between PA and depression prevalence, in order to establish the scope of the effect of PA on depressive symptoms and depression.

## 1. Introduction

Depression (major depressive disorder) is a common and serious medical illness that negatively affects how people feel, think and act. It causes feelings of sadness and/or a loss of interest in activities that once were enjoyed. It can lead to a variety of emotional and physical problems, decreasing peoples’ ability to function at work and in their private lives [1]. It also has some physical implications, such as weight loss, marked tiredness or lack of energy [2].

Depression is a frequent condition worldwide; 3.8% of the general population, 5% of adults and 5.7% of adults over 60 years old suffer from it [2]. In 2020, 5.3% of the Spanish population over 15 years old reported having a depressive state [3]. Regarding sex prevalence, depression is more common among females than in males (7.2% vs. 3.2%, respectively in 2020) [2]. The same phenomenon is observed regarding the severity of the depressive symptoms; great differences are observed between sexes (mild: 10.5% in women vs. 6.3% in men; severe: 0.9% women vs. 0.3% men) [3]. The frequency of depressive symptomatology in women (16.32%) almost doubled men′s frequency (8.94%) in all degrees of severity in 2020 [3]. As age increases, depression prevalence does too, so among Spanish 15 year olds there is a 0–2% prevalence of depression, at 45–74 years old a 2–4%, and at 74–84 years old between 4–6% [4].

Depression can become a serious health problem, especially when it is recurrent and of moderate or severe intensity. It causes pain and affects daily activities of those who suffer from it, which in the worst case can lead to suicide [5,6,7].

There are different ways to treat this disease, from pharmacological treatments such as selective serotonin reuptake inhibitors-SSRIs, trazodone-mirtazapine, duloxetine, desvenlafaxine, bupropion, heterocyclic antidepressants, etc. [8], to non-pharmacological treatments such as acupuncture, omega-3 fatty acids, or psychotherapies [9,10], that entail high economic costs for the health care system. Establishing health policies that focus on the prevention of this disease, such as health promoting campaigns, advice on good practices and health promotion through physical activity (PA) programs, seem to be effective in reducing the risk of depression [11,12,13].

PA shows beneficial effects combating depression [14], maybe due to the release of endorphins produced during the activity which reduces anxiety, depression and stress levels [15,16]. Numerous studies support the beneficial effects of physical activity on depression, both in terms of prevention and treatment [17,18,19,20,21,22].

In this sense, strength training has been shown to reduce depressive symptoms among adults, regardless of health status [23]; guidelines for the optimal frequency and days per week to produce these improvements have yet to be set. The aim of this study was to relate depression and depressive symptoms of the Spanish adult population, according to the Personal Health Questionnaire Depression Scale (PHQ-8), with the frequency of PA, and the days per week performed of PA and strength training declared in the European Health Survey of Spain 2020 [3].

## 2. Materials and Methods

### 2.1. Study Design

Based on the data obtained by the Ministry of Health and the Spanish National Institute of Statistics (INE) during the dates between 15 July 2019 and 24 July 2020, a cross-sectional research methodology, of a descriptive correlational nature, based on a survey was carried out using the information obtained thanks to the European Health Survey of Spain 2020 (EESE 2020). The EESE 2020 is the third edition of a survey carried out by professionals accredited by the INE in collaboration with the Ministry of Health, which aims to provide information on the health of the Spanish population over 15 years of age, in order to plan and evaluate health-related actions [24].

### 2.2. Participants 

INE selected the participants that made up the initial sample of the EESE 2020, using a three-stage stratified sampling methodology, which included the following aspects: census sections (municipalities), main family dwellings (following a systematic sampling with random start) and persons to be surveyed (one per dwelling, selected using the Kish random system) [3]. As inclusion criteria, participants needed to have an age between 15 and 89 years old at the time the survey was conducted; thus, 922 persons aged over 89 were excluded. The final sample consisted of 21,150 participants: 10,024 men and 11,126 women, aged between 15 and 89 years.

### 2.3. Ethics

The use of the present data does not require the approval of an accredited Ethics Committee, in accordance with the Regulation (EU) 2016/679 of the European Parliament and of the Council of 27 April 2016 on the protection of natural persons with regard to the processing of personal data and on the free movement of such data, the current data is not considered confidential, since they have been obtained through public and anonymous files, in compliance with the Regulation that came into force on 25 May 2016 and is mandatory as of 25 May 2018.

### 2.4. Informed Consent 

For this research, an informed consent was not required, given that the data was obtained from public data that is freely accessible, anonymous and considered non-confidential. In EESE 2020, prior to the interviews, the selected participants were informed, by letter, of their inclusion in the survey and informed them of the confidential nature of the data collection and dissemination, and of the regulations that protect them.

### 2.5. Variables and Procedures

The study variables used in this research were constructed, based on the data extracted from the responses of the participants in the EESE 2020. These variables are expressed next: 

Sex (men and women), age (years), age group (derived from the previous variable, participants were grouped into: 15–34 years, 35–49 years, 50–64 years, 65–74 years and 75–89 years).

Social class: this variable was defined in the EESE2020 methodology. Participants were grouped into: Class I (directors and managers of establishments with 10 or more employees and professionals traditionally associated with university degrees), Class II (directors and managers of establishments with fewer than 10 employees, professionals traditionally associated with university degrees and other technical support professionals, and sportsmen and sportswomen), Class III (intermediate occupations and self-employed workers), Class IV (supervisors and workers in skilled technical occupations), Class V (skilled workers in the primary sector and other semi-skilled workers) and Class VI (unskilled workers).

The frequency of PA (derived from the answers given to question p.112 of the EESE 2020, “Which of these possibilities best describes the frequency with which you do PA in your free time?”, participants were grouped into: never (I do not exercise. I spend my free time almost completely sedentary), occasional (I do some occasional PA or sports), several/month (I do PA several times a month); several/week (I do sports or physical training several times a week); nineteen participants were excluded for the analyses that included this variable because they answered “Don’t know” or “No answer” (DK/NA).

PA days (derived from responses given to EESE 2020 question p.117, “In a week of normal activity how many days do you practice sports, gymnastics, cycling, brisk walking, etc., at least 10 min at a time?”, participants were grouped into: 0 days/week, 1–2 days/week, 3–4 days/week and 5+ days/week. For the analyses including this variable, 33 participants who answered “DK/NA” were excluded).

Strength training days (derived from responses given to question p.119 of the EESE 2020, “In a week of normal activity how many days do you do activities specifically aimed at strengthening your muscles?”. Participants were grouped into: 0 days/week, 1–2 days/week, 3–4 days/week and 5+ days/week).

Depressive symptoms (derived from the variable Depressive Severity, included in the EESE 2020, constructed from questions p.41.A–p.47.H, corresponding to the phq-8 questionnaire, adding the values from 0 to 3, according to the answers given.

The measures used to evaluate the data have high quality, since it is an objective questionnaire (because it responds to what the participants select in the questionnaire, whether in the personal interview or in the telephone interview), it is reliable (since the measurement through the questionnaire is the same for the entire sample) and valid (since it will measure health indicators to determine information on the health of the Spanish population) [24]. Additionally, the validity of the phq-8 questionnaire has been proven [25,26,27].

In the EESE 2020, participants were classified according to their degree of depression into the following groups: None (scores below 5), Mild (scores between 5 and 9), Moderate (scores between 10 and 14), Moderately Severe (scores between 15 and 19) and Severe (scores above 19). For this research, participants were grouped into: None, Mild/Moderate and Moderately Severe/Serious. Symptoms (derived from the previous variable, grouped into: Yes (Mild/Moderate and Moderately Severe/Serious) and No (None)). Depression (derived from the variable “Cuadros depresivos”, included in the ENSE 2020, which is constructed from questions p.41.A–p.47.H, that are part of the phq-8 questionnaire, according to the appearance of depressive symptoms and its duration. The groupings used in the EESE 2020 were used, renamed to: Major Depression (participants were grouped into this variable, if they had five or more symptoms (one of them being anhedonia or depression), more than half of the days), Other Depression (if they had between 2–4 symptoms, more than half of the days, including depressed mood or anhedonia), None (less than two symptoms) and Depression (derived from the previous variable, grouping into: Yes (Major Depression, or Other Depression) and No (none) [28].

### 2.6. Statistical Analysis 

IBM SPSS Statistics v.25 software was used to perform the statistical analysis of this research.

After performing the Kolgomorov-Smirnov test, sufficient evidence was not found to assume that the data followed a normal distribution. Therefore, a descriptive analysis was performed, presenting the data of the variables through the median and the interquartile range (age, being a continuous variable) and the absolute and relative frequencies (sex, age group, frequency of PA, days of PA, days of strength, depression and depressive symptoms, being ordinal variables). To analyze possible intergroup differences, nonparametric statistical tests were used: Mann-Whitney U test (to analyze intergroup differences in continuous variables: age), chi-square statistic and Levene’s z test (to find dependency relationships and analyze possible differences between proportions, respectively, in the analysis of ordinal variables: sex, age group, frequency of PA, days of PA, days of strength, depression and depressive symptoms, symptoms and depression), effect size was evaluated with contingency coefficient. A binary multiple logistic regression analysis was performed, taking as dependent variables: depression symptoms; depression; and as independent variables: sex, age, social class and PAL

For all these analyses, a significance level of less than 0.05 was established.

## 3. Results

Regarding age, the median of the population studied was 55 years; it was significantly lower in men (53) than in women (56) (*p* < 0.001). Dependency relationships were found between age group and sex (*p* < 0.001) (Table 1). 

Dependency relationships were found between sex and prevalence of depressive symptoms (*p* < 0.001). Prevalence of major depression was higher in women than in men (3.8% vs. 1.6%, *p* < 0.05), for the general population a 2.8% prevalence was found. In the general population, 3.4% presented depression, differences between women and men were found (4.3% vs. 2.2%, *p* < 0.05). As with depression, dependency relationships were found between sex and depressive symptoms (*p* < 0.001). Moderately severe or severe depressive symptoms had higher prevalence in women than in men, being 2.7% in women and 1.1% in men; mild or moderate depressive symptoms had a prevalence of 15.3% in women and 8.6% in men; these differences were significant in both depressive symptom types (*p* < 0.05).

In total, 75.5% of the general population reported not performing PA regularly (36.6% Never, 38.9% Occasionally), 10.2% reported performing PA several times a month and 14.4%, several times a week. The dependency relationships were found between PA frequency and sex (*p* < 0.001). The proportion of inactive participants was higher in women than in men (Never: 40.0% vs. 32.8%, *p* < 0.05), the percentage of active men (11.6%, several times a month and 16.6%, several times a week) was higher than that of women (8.9% and 12.3%, respectively), these differences were significant (*p* < 0.05).

Dependency relationships were also found between days per week of PA and sex (*p* < 0.001). In the general population, 52.9% did not perform PA any day per week; this proportion was higher in women (55.8%) than in men (49.7%), these differences were significant (*p* < 0.05). The proportion of participants who performed PA 3 days or more per week were higher in men than in women, 17.5% vs. 15.4%, that performed 3–4 days per week; and 21.0% vs. 16.5%, with 5 or more days performed per week (*p* < 0.05 in both cases).

Regarding strength training, the percentage of people who never performed this type of activity was even higher; it was found that 82.5% of the general population never performed strength training not performing strength training any day of per week. Significant differences between the proportion of men and women that performed strength training was found (84.7% vs. 80.1%, *p* < 0.05). Dependence relationships between days of strength training and sex were found (*p* < 0.001). 

**Figure 1:**
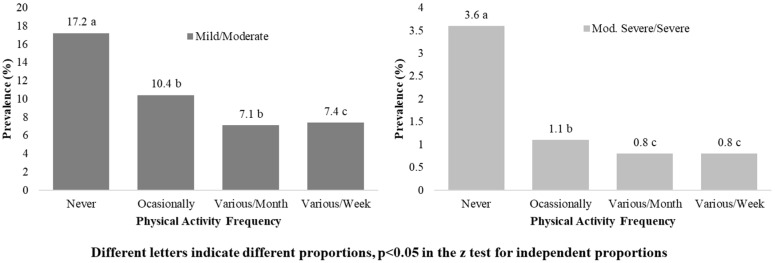
PA frequency and prevalence of depressive symptoms in the general population.

Dependency relationships were found between the frequency of PA and depressive symptoms, in the general population and in both sexes too (*p* < 0.001). Figure 1 shows the prevalence of mild and moderate depressive symptoms, and moderately severe and severe depressive symptoms, in the different groups of PA frequency of the general population. Higher prevalence was found in the inactive population than in the rest of PA frequencies (*p* < 0.05). Similar results were found in men and women, as shown in Table 2. Regarding mild and moderate symptoms, prevalence went from 20.5% in women who never performed PA to 8.8% in women with several activities per month, where *p* < 0.05. In men, from a prevalence of 12.8% in inactive participants to 5.5% in men with several activities per week, where *p* < 0.05. For moderately severe or severe depressive symptoms, similar results were found.

**Figure 2:**
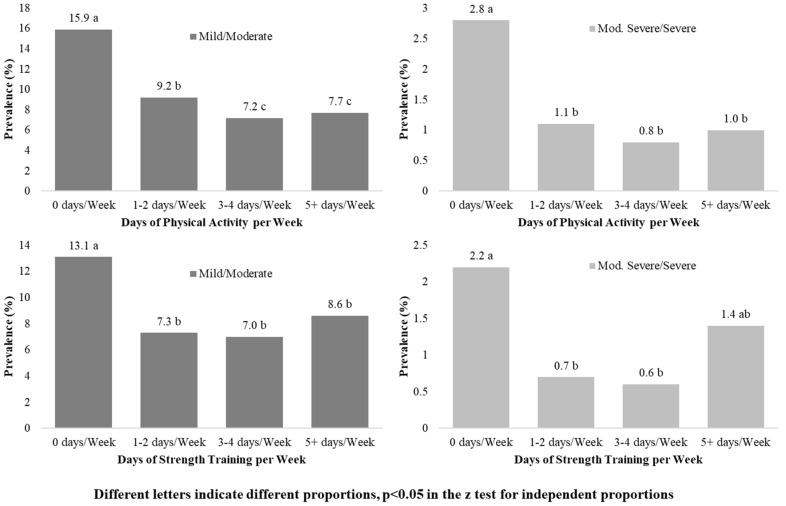
PA, strength training and prevalence of depressive symptoms in the general population.

Regarding days of PA per week, dependency relationships were found between PA frequency and depressive symptoms, and PA frequency and sex, in the general population (*p* < 0.001). Figure 2 shows the prevalence of depressive symptoms (mild and moderate, and moderately severe and severe) in the general population, classified according to the number of days per week that they performed PA and strength training. The prevalence of mild and moderate, and moderately severe and severe depressive symptoms was higher in people who reported not performing PA and strength training any days per week, and lower in people who performed these activities between 3 and 4 days, with significant differences between both proportions (*p* < 0.05). Grouping by sex, those men who did not perform any PA days per week had the highest prevalence of mild or moderate depressive symptoms (11.5%), the lowest prevalence was found in those who performed PA 3–4 days per week (4.9%), *p* < 0.05. Regarding strength training, the highest prevalence of depressive symptoms was found in those who did not perform this exercise any day per week (9.3%), and the lowest in those who performed it 3–4 days a week (4.5%) (*p* < 0.05). In women, the highest prevalence of mild or moderate symptoms was found, both in women who did not perform PA any day a week (19.4%), and in those who did not perform strength training (16.4%). As in men, the lowest prevalence was found among those who performed PA 3–4 days a week (9.5%), with *p* < 0.05 compared to those who did not perform PA 3–4 days a week. However, the lowest prevalence, regarding strength training frequency was found in those who performed it 1–2 days per week (6.0%), with *p* < 0.05, compared to those who did not perform it any day. Similar results were found when comparing the prevalence of moderately severe and very severe symptoms with PA and strength training days. 

**Figure 3:**
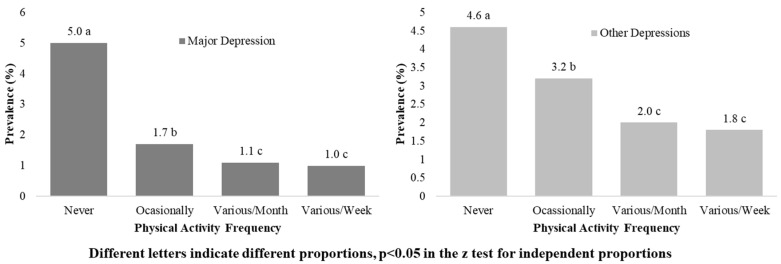
Prevalence of major depression and other types of depression, according to frequency of PA in the general population.

Dependency relationships were found between PA frequency and depressive symptoms, in the general population and by sexes too (*p* < 0.001). For the general population, the prevalence of major depression was lower in the active groups than in the inactive population: 5.0% (never), 1.7% (occasionally), 1.1% (several times a month) and 1.0% (several times a week); something similar to what was found in the prevalence of other forms of depression (Figure 3). The same dependency relationships were found in both sexes (*p* < 0.001) (Table 3).

In both sexes the prevalence of major depression was higher in the inactive participant group, with differences in proportions with the rest of the groups (*p* < 0.05). The prevalence of depression, according to the frequency of PA was in women: 6.3% (never), compared to 1.5% (several times a week), with *p* < 0.05, and in men: 3.2% (never), versus 0.4% (several times a month), with *p* < 0.05.

**Figure 4:**
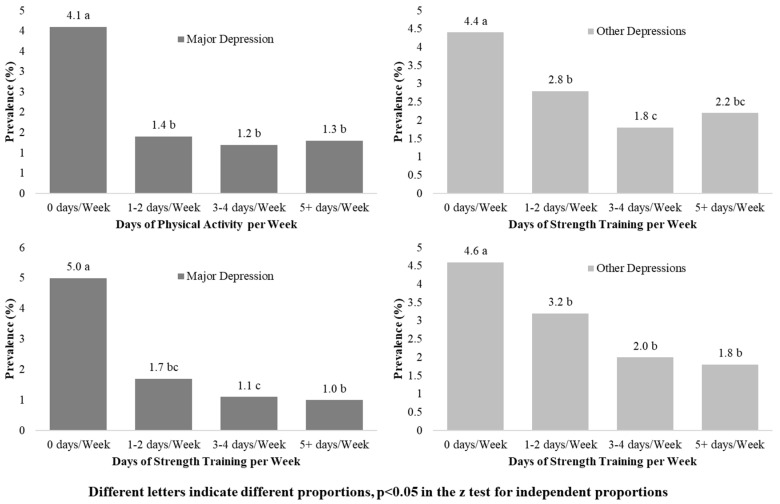
Prevalence of major depression and other types of depression, according to the PA and strength training days performed a week.

In addition, dependency relationships were found between the days per week of PA performed and depression, as well as between the number of performed strength training days and depression, these occurred in the general population (Figure 4) and in both sexes (*p* < 0.001). The highest prevalence of major depression and other types of depressions was found in the groups that did not perform at least one day of PA or strength training activities.

Table 4 shows the relationships found between depressive symptoms and frequency of PA, days per week of PA and strength training, in different age groups. As well as the differences in proportions that were found between the different PA groups. Except in the youngest age group (18–34 years), significant dependency relationships were found between presenting any depressive symptom and the PA variables, with *p* < 0.001 (for frequency of PA and days of PA per week) and *p* < 0.05 (for days of strength training, except in the 35–49 age group, with *p* < 0.001).

Something similar was found when analyzing the PA and depression variables in each age group. Presenting some type of depressive disorder was related to the frequency of PA in all age groups (*p* < 0.001), except in the 18–34 years group (*p* < 0.001). The same was observed between depressive disorder and PA days (Table 5), with dependency relationships in all age groups: 18–34 years (*p* < 0.005) and all other groups (*p* < 0.001). In contrast, there was no clear evidence of such relationships between suffering any depressive condition and strength training days performed per week in all age groups. The highest prevalence of depressive symptoms was found in those who did not perform PA at any time; this occurred in all groups and for all PA variables.

Table S1 shows the binary multiple logistic regression model, explaining 9.1% of the variance (Nagelkerke’s R^2^) of the depression symptoms variable.

Table S2 shows the binary multiple logistic regression model, explaining 8.5% of the variance (Nagelkerke’s R^2^) of depression.

## 4. Discussion

The present results highlight gender as an important factor in relation to depressive symptoms. Female gender is associated with a higher prevalence of depression than male in all age groups [3,29,30]. These sex differences may have influenced the results. There are different theories that try to justify why women suffer more from depression. Zarragoitía (2013) [31] proposed that women are more prone to suffer depression due to biological factors like hormonal changes, pregnancy or in old age: menopause or factors attributed to physical problems. Bebbington (1998) [32] attributes this to social and structural factors, since being widowed makes you more prone to depression, having a greater number of children is associated with a higher level of depression, and a lower level of education means higher prevalence of depression [32]. However, one of the hypotheses with more evidence suggests that women have higher levels of depression due to the social, legal and economic discrimination they suffer, which leads to states of helplessness, dependence on others, low aspirations and low self-esteem that end up causing depressive symptoms and depression [33].

Regarding age, it was found that as age increases depressive symptoms increase, reaching a maximum between the ages of 50–64 years. In fact, Montesó (2014) [34] reported that from the age of 65 years depression increases in women, although this study had some limitations as a small sample and it was limited only to a specific geographical area of Spain. Regarding women, it could also be explained by menopause occurring at these ages [35,36]. 

The main finding of the present study was the dependent relationships found between the prevalence of depression and PA frequency, in the general population and by sex. Depression prevalence was higher in the inactive groups, decreasing in the groups with higher PA frequency. Several studies support these results [15,16,37,38], stating that is mandatory to increase the importance of PA to reduce the likelihood of depression. Rosenbaum (2014) [22] reported that PA reduces depressive symptoms in people with mental illness. Cornejo (2017) [39] concluded that exercise is an effective treatment for mild and moderate depression [39].

Dependence relationships were found between the prevalence of depression and the days per week of PA and strength training, both in the general population, by sex and different age groups. These results concorded with previous systematic reviews on physical exercise and depression [40,41]. At older ages, a higher frequency of strength training would be required to decrease the prevalence of depression, which agrees with the findings of Gordon et al., (2018) [23]. In addition, it has been shown that the incorporation of strength training exclusively or combined with aerobic training decreases the prevalence of depression [14,42]. Regarding frequency, there are no exact guidelines for the treatment of depression through exercise, since volume, intensity and frequency are parameters that will influence training as well as the chosen training modality [42,43,44,45]. 

### 4.1. Practical Applications

Looking to the future, it should be noticed form this study, that new policies are needed to promote PA throughout the country. Some examples of these policies could be guides and advice for families about PA, sports programs promoted by municipalities, educational centers and companies, policies to promote PA as a way of transportation, creating areas for the practice of PA, active aging programs, etc. These policies are aimed to increase the frequency of PA in all age groups, as inactivity has a potential risk for depression [15,16], and as we know, depression can appear at any age [46].

At older ages it would be important to increase training frequency to obtain greater benefits in terms of depression, at least 1–2 days per week (although the training frequency should be adjusted according to age). In addition, PA should be complemented with strength training programs, which have been shown to be effective in reducing depression [14,42,47]. According to the recommendations of the American College of Sports Medicine (2020) [48], 2–3 days a week of strength training are needed for it to be effective at attenuating depression. As the recommended frequency of PA and the type of exercise to prevent or reduce the prevalence of depression for different age and sex groups are known, our results could serve as a reference for developing prevention strategies for the Spanish population.

### 4.2. Limitations

This research has some limitations that should be taken in consideration; since it is a cross-sectional study, where data was obtained from the public files of the EESE 2020, it is not possible to establish causal relationships. It would be interesting to make other research designs that would allow to establish such relationships as future lines. Additionally, the sample only considered male and female for the sex variable, not including non-binary sex. Data on the consumption of antidepressants and other variables that may affect depression, such as sociodemographic, socioeconomic or sociocultural variables was not collected, since this could mean a reduction in the sample if we divided it in these groups.

## 5. Conclusions

There exist dependency relationships between the frequency of PA and the prevalence of depressive symptoms and depression, as well as between these and the number of days per week of PA and strength training, both in the general population, by sex and in the different age groups. The worst results for depressive symptoms and depressive conditions are found in the inactive population.

Future studies are required to establish the exact relationship between PA and depression prevalence. A wider body of evidence is required to define the exact scope of PA on depression and depressive symptoms. If this relationship is confirmed, guidelines on how to perform PA in order to positively affect depressive disorder would need to be established.

## Figures and Tables

**Table 1:** Characterization of the Spanish population aged 18 to 89 years from the EESE2020, and gender differences.

Variables				
Age (years)	Total (*n* = 21,150)	Men (*n* = 10,024)	Women (*n* = 11,126)	*p*
Median (IQR)	55 (27)	53 (26)	56 (28)	<0.001
Mean (SD)	54.7 (17.7)	53.7 (17.2)	55.7 (18.1)	-
Age group (years)	Total (*n* = 21,150)	Men (*n* = 10,024)	Women (*n* = 11,126)	p*
18–34	2913 (13.8)	1409 (14.1)	1504 (13.5)	<0.001
35–49	5647 (26.7)	2841 (28.3)	2806 (25.2)	
50–64	5842 (27.6)	2881 (28.7)	2961 (26.6)	
65–74	3404 (16.1)	1555 (15.5)	1849 (16.6)	
75–89	3344 (15.8)	1338 (13.3)	2006 (18.0)	
PA frequency	Total (*n* = 21,131)	Men (*n* = 10,014)	Women (*n* = 11,117)	p*
Never	7733 (36.6)	3282 (32.8)	4451 (40.0)	<0.001
Occasionally	8212 (38.9)	3906 (39.0)	4306 (38.7)	
Various/Month	2153 (10.2)	1163 (11.6)	990 (8.9)	
Various/Week	3033 (14.4)	1663 (16.6)	1370 (12.3) *	
PA frequency (days per week)	Total (*n* = 21,017)	Men (*n* = 9965)	Women (*n* = 11,052)	p*
0 days/week	11,122 (52.9)	4953 (49.7)	6169 (55.8) *	<0.001
1–2 days/week	2535 (12.1)	1180 (11.8)	1355 (12.3)	
3–4 days/week	3447 (16.4)	1743 (17.5)	1704 (15.4) *	
5+ days/week	3913 (18.6)	2089 (21.0)	1824 (16.5) *	
Strength training days	Total (*n* = 20,959)	Men (*n* = 9933)	Women (*n* = 11,026)	p*
0 days/week	17,291 (82.5)	7953 (80.1)	9338 (84.7) *	<0.001
1–2 days/week	1470 (7.0)	682 (6.9)	788 (7.1)	
3–4 days/week	1395 (6.7)	816 (8.2)	579 (5.3) *	
5+ days/week	803 (3.8)	482 (4.9)	321 (2.9) *	
Depressive Symptoms	Total (*n* = 21,013)	Men (*n* = 9961)	Women (*n* = 11,052)	p*
None	18,065 (86.0)	8996 (90.3)	9069 (82.1) *	<0.001
Mild/Moderate	2541 (12.1)	854 (8.6)	1687 (15.3) *	
Moderate Severe/Severe	407 (1.9)	111 (1.1)	296 (2.7) *	
Depression	Total (*n* = 21,013)	Men (*n* = 9961)	Women (*n* = 11,052)	p*
Major Depression	579 (2.8)	163 (1.6)	416 (3.8) *	<0.001
Other Depression	706 (3.4)	231 (2.2)	475 (4.3) *	
None	19,728 (93.9)	9567 (96.0)	10,161 (91.9) *	

IQR (interquartile range); SD (standard deviation); data presented in absolute and relative frequencies (ordinal variables); *p* (*p*-value from Mann-Whitney U test); p* (*p*-value from chi-square test); * (significant differences between proportions of men and women at 95% z-test for independent proportions); Major Depression (five or more presenting symptoms); Other Depression (2–4 symptoms); None (fewer than 2 symptoms); None (scores below 5 on phq8); Mild/Moderate (scores between 5 and 14 on phq8); Moderate Severe/Severe (scores above 15 on phq8); Never (state: “I do not exercise. I spend my free time almost completely sedentary in item 112 (Which of these possibilities best describes how often you do any PA in your free time?)); Occasionally (“I do some occasional PA or sports” in item 112); Several times a month (“I do PA several times a month” in item 112); Several times a week (“I do sports or physical training several times a week” in item 112); days/week (response to item: 117 (how many days do you practice sports, gymnastics, cycling, brisk walking, etc., at least 10 min at a time?); 119 (how many days do you do activities specifically aimed at strengthening your muscles?).

**Table 2:** Relationship between frequency of PA, days of activity and muscle strengthening per week and depressive symptoms.

Pa Frequency							
General Population							
Depressive Symptoms	Never	Occasional	Various/Month	Various/Week	Total	*p*	Eff. Size
None	6057 (79.2) a	7234 (88.5) b	1979 (92.1) c	2782 (91.8) c	18,052 (86.0)	<0.001	0.156
Mild/Moderate	1317 (17.2) a	847 (10.4) b	152 (7.1) c	224 (7.4) c	2540 (12.1)		
Moderate Severe/Severe	272 (3.6) a	94 (1.1) b	17 (0.8) b	23 (0.8) b	19,714 (93.9)		
Men							
Depressive Symptoms	Never	Occasional	Various/month	Various/week	Total	*p*	Eff. Size
None	2764 (85.0) a	3569 (92.0) b	1090 (94.1) c	1565 (94.1) c	8988 (90.3)	<0.001	0.132
Mild/Moderate	415 (12.8) a	281 (7.2) b	65 (5.6) b,c	92 (5.5) c	853 (8.6)		
Moderate Severe/Severe	72 (2.2) a	30 (0.8) b	3 (0.3)	6 (0.4) b	111 (1.1)		
Women							
Depressive Symptoms	Never	Occasional	Various/month	Various/week	Total	*p*	Eff. Size
None	3293 (74.9) a	3665 (85.3) b	889 (89.8) c	1217 (89.1) c	9064 (82.1)	<0.001	0.156
Mild/Moderate	902 (20.5) a	566 (13.2) b	87 (8.8) c	132 (9.7) c	1687 (15.3)		
Moderate Severe/Severe	200 (4.6) a	64 (1.5) b	14 (1.4) b	17 (1.2) b	295 (2.7)		
Days of Pa Per Week							
General Population							
Depressive Symptoms	0 days	1–2 days	3–4 days	5+ days	Total	*p*	Eff. Size
None	8948 (81.3) a	2274 (89.7) b	3169 (92.1) c	3562 (91.3) c	17,953 (86.0)	<0.001	0.143
Mild/Moderate	1746 (15.9) a	233 (9.2) b	247 (7.2) c	300 (7.7) c	2526 (12.1)		
Moderate Severe/Severe	313 (2.8)	27 (1.1) b	26 (0.8) b	39 (1.0) b	405 (1.9)		
Men							
Depressive Symptoms	0 days	1–2 days	3–4 days	5+ days	Total	*p*	Eff. Size
None	4253 (86.8) a	1099 (93.1) b	1649 (94.8) b	1941 (93.3) b	8942 (90.3)	<0.001	0.121
Mild/Moderate	563 (11.5) a	76 (6.4) b	85 (4.9) b	126 (6.1) b	850 (8.6)		
Moderate Severe/Severe	86 (1.8) a	5 (0.4) b	6 (0.3) b	14 (0.7) b	111 (1.1)		
Women							
Depressive Symptoms	0 days	1–2 days	3–4 days	5+ days	Total	*p*	Eff. Size
None	4695 (76.9) a	1175 (86.8) b	1520 (89.3) c	1621 (89.1) c	9011 (82.1)	<0.001	0.151
Mild/Moderate	1183 (19.4) a	157 (11.6) b	162 (9.5) b	174 (9.6) b	1676 (15.3)		
Moderate Severe/Severe	227 (3.7) a	22 (1.6) b	20 (1.2) b	25 (1.4) b	294 (2.7)		
Days of Strength Training Per Week							
General Population							
Depressive Symptoms	0 days	1–2 days	3–4 days	5+ days	Total	*p*	Eff.Size
None	14,539 (84.7) a	1349 (92.0) b	1286 (92.3) b	719 (90.0) b	17,893 (85.9)	<0.001	0.078
Mild/Moderate	2253 (13.1) a	107 (7.3) b	98 (7.0) b	69 (8.6) b	2527 (12.1)		
Moderate Severe/Severe	375 (2.2) a	11 (0.7) b	9 (0.6) b	11 (1.4) a,b	406 (1.9)		
Men							
Depressive Symptoms	0 days	1–2 days	3–4 days	5+ days	Total	*p*	Eff. Size
None	7064 (89.5) a	637 (93.7) b,c	774 (95.1) c	437 (91.0) a,b	8912 (90.3)	<0.001	0.063
Mild/Moderate	733 (9.3) a	41 (6.0) b,c	37 (4.5) c	37 (7.7) b,c	848 (8.6)		
Moderate Severe/Severe	100 (1.3) a	2 (0.3) b	3 (0.4) b	6 (1.3) a,b	111 (1.1)		
Women							
Depressive Symptoms	None	1–2 days	3–4 days	5+ days	Total	*p*	Eff. Size
None	7475 (80.6) a	712 (90.5) b	512 (88.4) b	282 (88.4) b	8981 (82.0)	<0.001	0.083
Mild/Moderate	1520 (16.4) a	66 (8.4) b	61 (10.5) b	32 (10.0)	1679 (15.3)		
Moderate Severe/Severe	275 (3.0) a	9 (1.1) b	6 (1.0) b	5 (1.6) a,b	295 (2.7)		

Data presented in absolute and relative frequencies (ordinal variables); *p* (*p*-value from chi-square test); abc (Each subscript corresponds to significant differences between column proportions at 95%); None (scores below 5 on phq8); Mild/Moderate (scores between 5 and 14 on phq8); Moderate Severe/Severe (scores above 15 on phq8); Never (they state: “I do not exercise. I spend my free time almost completely sedentary in item 112 (which of these possibilities best describes how often you do any PA in your free time?)); Occasional (“I do some occasional PA or sports” in item 112); Various/month (“*I do PA several times a month*” in item 112); Several/week (“I do sports or physical training several times a week” in item 112); days/week (response to item: 117 (how many days do you practice sport, gymnastics, cycling, brisk walking, etc., at least 10 min at a time?); 119 (how many days do you do activities specifically aimed at strengthening your muscles?). Eff. size (effect size: interpreted by contingency coefficient).

**Table 3:** Relationship between frequency of PA, days of activity and muscle strengthening a week and depression.

Pa Frequency							
General Population							
Depression	Never	Occasional	Various/month	Various/week	Total	*p*	Effect Size
Major Depression	382 (5.0) a	142 (1.7) b	24 (1.1) c	30 (1.0) c	578 (2.8)	<0.001	0.159
Other Depression	350 (4.6) a	260 (3.2) b	42 (2.0) c	54 (1.8) c	706 (3.4)		
None	6914 (90.4) a	7773 (95.1) b	2082 (96.9) c	2945 (97.2) c	19,714 (93.9)		
Men							
Depression	Never	Occasional	Various/month	Various/week	Total	*p*	Effect size
Major Depression	105 (3.2) a	43 (1.1) b	5 (0.4) c	10 (0.6) b,c	163 (1.6)	<0.001	0.136
Other Depression	104 (3.2) a	99 (2.6) b	16 (1.4) b	12 (0.7) b	231 (2.3)		
None	3042 (93.6) a	3738 (96.3) b	1137 (98.2) c	1641 (98.7) c	9558 (96.0)		
Women							
Depression	Never	Occasional	Various/month	Various/week	Total	*p*	Effect size
Major Depression	277 (6.3) a	99 (2.3) b	19 (1.9) b	20 (1.5) b	415 (3.8)	<0.001	0.165
Other Depression	246 (5.6) a	161 (3.7) b	26 (2.6) b	42 (3.1) b	475 (4.3)		
None	3872 (88.1) a	4035 (93.9) b	945 (95.5) b,c	1304 (95.5) c	10,156 (91.9)		
Days of Pa Per Week							
General Population							
Depression	0 days	1–2 days	3–4 days	5+ days	Total	*p*	Effect size
Major Depression	449 (4.1) a	35 (1.4) b	41 (1.2) b	50 (1.3) b	575 (2.8)	<0.001	0.146
Other Depression	488 (4.4) a	72 (2.8) b	61 (1.8) c	84 (2.2) b,c	705 (3.4)		
None	10,070 (91.5) a	2427 (95.8) b	3340 (97.0) c	3767 (96.6) b,c	19,604 (93.9)		
Men							
Depression	0 days	1–2 days	3–4 days	5+ days	Total	*p*	Effect size
Major Depression	131 (2.7) a	5 (0.4) b	9 (0.5) b	18 (0.9) b	163 (1.6)	<0.001	0.124
Other Depression	159 (3.2) a	27 (2.3) a,b	11 (0.6) c	34 (1.6) b	231 (2.3)		
None	4612 (94.1) a	1148 (97.3) b	1720 (98.9) c	2029 (b)	9558 (96.0)		
Women							
Depression	0 days	1–2 days	3–4 days	5+ days	Total	*p*	Effect size
Major Depression	318 (5.2) a	30 (2.2) b	32 (1.9) b	32 (1.8) b	412 (3.8)	<0.001	0.155
Other Depression	329 (5.4) a	45 (3.3) b	50 (2.9) b	50 (2.7) b	474 (4.3)		
None	5458 (89.4) a	1279 (94.5) b	1620 (95.2) b	1738 (95.5) b	10,095 (91.9)		
Days of Strength Training Per Week							
General Population							
Depression	0 days	1–2 days	3–4 days	5+ days	Total	*p*	Effect size
Major Depression	538 (3.1) a	14 (1.0) b,c	11 (0.8) c	14 (1.8) b	577 (2.8)	<0.001	0.079
Other Depression	637 (3.7) a	24 (1.6) b	25 (1.8) b	19 (2.4) b	705 (3.4)		
None	15,992 (93.2) a	1429 (97.4) b	1357 (97.4) b	766 (95.9) c	19,544 (93.8)		
Men							
Depression	0 days	1–2 days	3–4 days	5+ days	Total	*p*	Effect size
None	7064 (89.5) a	637 (93.7) b,c	774 (95.1) c	437 (91.0) a,b	8912 (90.3)	<0.001	0.065
Mild/Moderate	733 (9.3) a	41 (6.0) b,c	37 (4.5) c	37 (7.7) b,c	848 (8.6)		
Moderate Severe/Severe	100 (1.3) a	2 (0.3) b	3 (0.4) b	6 (1.3) a,b	111 (1.1)		
Women							
Depression	None	1–2 days	3–4 days	5+ days	Total	*p*	Effect size
Major Depression	389 (4.2) a	11 (1.4) b	6 (1.0) b	8 (2.5) a,b	414 (3.8)	<0.001	0.086
Other Depression	434 (4.7) a	12 (1.5) b	19 (3.3) a	9 (2.8) a,b	474 (4.3)		
None	8447 (91.1) a	764 (97.1) b	554 (95.7) b	302 (94.7) b	10,067 (91.9)		

Data presented in absolute and relative frequencies (ordinal variables); *p* (*p*-value from chi-square test); abc (each subscript corresponds to significant differences between column proportions at 95% z-test for independent proportions); Major Depression (five or more symptoms presented); Other Depression (2–4 symptoms); None (fewer than 2 symptoms); Never (they state: “I do not exercise. My free time is spent almost entirely sedentary in item 112 (which of these possibilities best describes how often you do any PA in your free time?)); Occasional (“I do some occasional PA or sports” in item 112); Several/month (“I do PA several times a month” in item 112); Several/week (“I do sports or physical training several times a week” in item 112); days/week (response to item: 117 (how many days do you practice sport, gymnastics, cycling, brisk walking, etc., at least 10 min at a time?); 119 (how many days do you do activities specifically aimed at strengthening your muscles?); effect size (interpreted by contingency coefficient).

**Table 4:** Relationship between the frequency of PA. Days a week of PA and muscle strengthening and having any depressive symptoms, according to phq8 in the Spanish population by age group.

Pa Frequency						
Age Group	Symptoms	Never	Occasional	Various/Month	Various/Week	*p*
18–34 years	Yes	77 (8.9) a	65 (7.3) a	29 (6.3) a	53 (7.7) a	0.376
35–49 years	Yes	236 (12.6) a	190 (9.7) b	58 (7.7) b,c	72 (6.9) c	<0.001
50–64 years	Yes	384 (19.0) a	305 (12.2) b	49 (9.1) c	73 (9.7) b,c	<0.001
65–74 years	Yes	282 (24.4) a	191 (12.0) b	22 (8.5) b	33 (8.8) b	<0.001
75–89 years	Yes	610 (35.2) a	190 (15.4) b	11 (7.8) c	16 (9.3) c	<0.001
Pa Days Per Week						
Age Group	Symptoms	None	1–2 days	3–4 days	5+ days	*p*
18–34 years	Yes	106 (9.3) a	30 (6.7) a,b	42 (5.5) b	45 (8.4) a	<0.05
35–49 years	Yes	333 (13.1) a	79 (8.5) b	76 (6.3) b	67 (7.2) b	<0.001
50–64 years	Yes	520 (17.3) a	79 (10.3) b	85 (9.4) b	122 (11.0) b	<0.001
65–74 years	Yes	381 (20.1) a	47 (18.4) a	44 (11.5) b	54 (6.5) c	<0.001
75–89 years	Yes	719 (29.7) a	25 (18.4) b	26 (13.8) b,c	51 (10.1) c	<0.001
Strength Training Days Per Week						
Age Group	Symptoms	None	1–2 days	3–4 days	5+ days	*p*
18–34 years	Yes	160 (8.4) a	19 (5.4) a	25 (5.9) a	19 (9.0) a	0.085
35–49 years	Yes	461 (10.9) a	37 (6.4) b	36 (7.0) b	21 (8.6) a,b	<0.001
50–64 years	Yes	719 (14.5) a	39 (11.0) a,b	29 (10.1) b	21 (11.5) a,b	<0.05
65–74 years	Yes	485 (16.3) a	17 (12.8) a,b	12 (9.4) b	11 (9.9) a,b	<0.05
75–89 years	Yes	803 (25.8) a	6 (12.8) b	5 (13.9) a,b	8 (15.1) a,b	<0.05

Data presented in absolute and relative frequencies; *p* (*p*-value from the chi-square test); abc (each subscript corresponds to significant differences between column proportions at 95%); symptoms (depressive symptoms, according to phq8); Yes (scores greater than 5 on phq8); Never (they state: “I do not exercise. My free time is spent almost completely sedentary in item 112 (Which of these possibilities best describes how often you do any PA in your free time?)); Occasional (“I do some occasional PA or sports” in item 112); several/month (“I do PA several times a month” in item 112); several/week (“I do sports or physical training several times a week” in item 112); days/week (response to item: 117 (how many days do you practice sport. gymnastics, cycling, brisk walking, etc., at least 10 min at a time); 119 (how many days do you do activities).

**Table 5:** Relationship between the frequency of PA. Days week of PA and muscle strengthening and some type of diagnosed depression, according to phq8 in the Spanish population by age group.

Pa Frequency						
Age Group	Depression	Never	Occasional	Various/Month	Various/Week	*p*
18–34 years	Yes	32 (3.7) a	26 (2.9) a,b	7 (1.5) b	16 (2.3) a,b	0.114
35–49 years	Yes	101 (5.4) a	71 (3.6) b	23 (3.1) b	25 (2.4) b	<0.001
50–64 years	Yes	186 (9.2) a	136 (5.4) b	19 (3.5) b,c	23 (3.0) c	<0.001
65–74 years	Yes	135 (11.7) a	79 (5.0) b	11 (4.2) b	11 (2.9) b	<0.001
75–89 years	Yes	278 (16.1) a	90 (7.3) b	6 (4.3) b	9 (5.2) b	<0.001
Days of Pa Per Week						
Age group	Depression	None	1–2 days	3–4 days	5+ days	*p*
18–34 years	Yes	43 (3.8) a	17 (3.8) a	7 (0.9) b	14 (2.6) a	<0.005
35–49 years	Yes	137 (5.4)	33 (3.6) b	28 (2.3) b	21 (2.3) b	<0.001
50–64 years	Yes	252 (8.4) a	28 (3.6) b	36 (4.0) b	48 (4.3) b	<0.001
65–74 years	Yes	177 (9.3) a	15 (5.9) a,b	18 (4.7) b,c	26 (3.1) c	<0.001
75–89 years	Yes	328 (13.6) a	14 (10.3) a,b	13 (6.9) b,c	25 (5.0) c	<0.001
Strength Training Days Per Week						
Age group	Depression	None	1–2 days	3–4 days	5+ days	*p*
18–34 years	Yes	64 (3.4) a	4 (1.1) b	8 (1.9) a,b	5 (2.4) a,b	0.061
35–49 years	Yes	188 (4.4) a	10 (1.7) b	13 (2.5) b	9 (3.7) a,b	<0.005
50–64 years	Yes	331 (6.7) a	14 (3.9) b	10 (3.5) b	9 (4.9) a,b	<0.05
65–74 years	Yes	221 (7.4) a	7 (5.3) a,b	3 (2.3) b	5 (4.5) a,b	0.081
75–89 years	Yes	371 (11.9) a	3 (6.4) a	2 (5.6) a	5 (9.4) a	0.392

Data presented in absolute and relative frequencies (ordinal variables); *p* (*p*-value from chi-square test); abc (each subscript corresponds to significant differences between column proportions at 95%); Depression (depressive state detected by the phq8 questionnaire); Yes (2 or more symptoms detected in the phq8); Never (they state: “I do not exercise. I spend my free time almost completely sedentary in item 112 (which of these possibilities best describes the frequency with which you do some PA in your free time?)); Occasional (“I do some occasional PA or sports” in item 112); various/month (“I do PA several times a month” in item 112); Several/week (“I do sports or physical training several times a week” in item 112); days/week (response to item: 117 (how many days do you practice sports, gymnastics, cycling, brisk walking, etc., at least 10 min at a time?); 119 (how many days do you do activities specifically aimed at strengthening your muscles?).

## Data Availability

Data used were obtained from public use files, available on the Spanish Ministry of Health, Consumer Affairs, and Social Welfare website: https://www.sanidad.gob.es/estadEstudios/estadisticas/encuestaNacional/encuesta2017.htm (accessed on 3 May 2022). Additional datasets will be available upon reasonable request.

## Supplementary Materials

The following supporting information can be downloaded at: https://www.mdpi.com/article/10.3390/ijerph192214704/s1, Table S1: Logarithmic binary regression model for depression, Table S2: Logarithmic binary regression model for depression symptons.
