# Supplementary material for: Physical Activity Frequency and Depression in the Spanish Population
Source: Int J Environ Res Public Health. 2022 Nov 9;19(22):14704. doi: 10.3390/ijerph192214704 (PMC9691247; doi:10.3390/ijerph192214704)
Supplement: Supplementary file 1 [file ijerph-19-14704-s001.zip › ijerph-1953446-supplementary.pdf]

Table S1. Logarithmic binary regression model for depression.

	B	S.E.	Wald	df	Sig.	Exp(B)	95% C.I.for EXP(B)	
							Lower	Upper
Sex (Women)	-0.724	0.052	192.512	1	0.000	0.485	0.438	0.537
Social class (VI)			50.266	5	0.000			
I	-0.601	0.109	30.620	1	0.000	0.548	0.443	0.678
II	-0.495	0.115	18.502	1	0.000	0.610	0.486	0.764
III	-0.449	0.082	29.812	1	0.000	0.638	0.543	0.750
IV	-0.223	0.086	6.769	1	0.009	0.800	0.677	0.947
V	-0.221	0.070	9.861	1	0.002	0.801	0.698	0.920
Age	0.025	0.002	287.279	1	0.000	1.026	1.023	1.029
PA Frequency (Never)			25.143	3	0.000			
Occasional	-0.286	0.062	21.018	1	0.000	0.751	0.664	0.849
Various/month	-0.449	0.124	13.181	1	0.000	0.638	0.501	0.813
Various/week	-0.367	0.113	10.558	1	0.001	0.693	0.555	0.864
PA Days (None)			14.108	3	0.003			
1-2 days	0.046	0.099	0.213	1	0.644	1.047	0.862	1.272
3-4 days	-0.294	0.102	8.313	1	0.004	0.745	0.610	0.910
5+ days	-0.209	0.088	5.677	1	0.017	0.811	0.683	0.964
Strength training days (None)			10.612	3	0.014			
1-2 days	-0.447	0.146	9.423	1	0.002	0.639	0.481	0.851
3-4 days	0.074	0.141	0.276	1	0.599	1.077	0.817	1.419
5+ days	-0.133	0.175	0.580	1	0.446	0.875	0.621	1.234
Constant	-3.195	0.208	236.416	1	0.000	0.041		

B: Understandarized beta; SE: Standard error of the regression; Wald: Wald Chi-Squared Test; Df: Degrees of freedom; Sig: Statistical significance; Exp: Exponential regression; CI: Confidence Interval

Table S2. Logarithmic binary regression model for depression symptons.

	B	S.E.	Wald	df	Sig.	Exp(B)	95% C.I.for EXP(B)	
							Lower	Upper
Sex (Women)	-0.659	0.064	104.411	1	0.000	0.517	0.456	0.587
Social class (VI)			41.672	5	0.000			
I	-0.731	0.140	27.229	1	0.000	0.481	0.366	0.633
II	-0.635	0.148	18.289	1	0.000	0.530	0.396	0.709
III	-0.430	0.099	19.029	1	0.000	0.651	0.536	0.789
IV	-0.237	0.103	5.329	1	0.021	0.789	0.645	0.965
V	-0.293	0.085	11.978	1	0.001	0.746	0.632	0.881
Age	0.023	0.002	158.749	1	0.000	1.023	1.020	1.027
PA Frequency (Never)			38.657	3	0.000			
Occasional	-0.453	0.077	34.714	1	0.000	0.636	0.547	0.739
Various/month	-0.535	0.158	11.495	1	0.001	0.586	0.430	0.798
Various/week	-0.630	0.151	17.451	1	0.000	0.533	0.396	0.716
PA Days (None)			22.367	3	0.000			
1-2 days	-0.057	0.127	0.200	1	0.654	0.945	0.736	1.213
3-4 days	-0.401	0.133	9.095	1	0.003	0.670	0.516	0.869
5+ days	-0.499	0.119	17.573	1	0.000	0.607	0.481	0.767
Strength training days (None)			5.699	3	0.127			
1-2 days	-0.224	0.182	1.516	1	0.218	0.800	0.560	1.142
3-4 days	0.009	0.196	0.002	1	0.963	1.009	0.687	1.481
5+ days	0.393	0.203	3.771	1	0.052	1.482	0.996	2.204
Constant	-3.080	0.137	508.714	1	0.000	0.046		

B: Understandarized beta; SE: Standard error of the regression; Wald: Wald Chi-Squared Test; Df: Degrees of freedom; Sig: Statistical significance; Exp: Exponential regression; CI: Confidence Interval
